# Association of cyclooxygenase-2 expression with endoplasmic reticulum stress and autophagy in triple-negative breast cancer

**DOI:** 10.1371/journal.pone.0289627

**Published:** 2023-08-04

**Authors:** Haechan Lee, SungWook Jung, Gyungyub Gong, Bora Lim, Hee Jin Lee

**Affiliations:** 1 University of Ulsan College of Medicine, Seoul, Korea; 2 Department of Medical Science, AMIST, Asan Medical Center, University of Ulsan College of Medicine, Seoul, Republic of Korea; 3 Department of Pathology, Asan Medical Center, University of Ulsan College of Medicine, Seoul, Korea; 4 Department of Hematology and Oncology, Baylor College of Medicine, Houston, TX, United States of America; Columbia University, UNITED STATES

## Abstract

Cyclooxygenase-2 plays a role in oncogenesis and its overexpression is associated with triple-negative breast cancer. However, the mechanisms whereby cyclooxygenase-2 contribute to breast cancer are complex and not well understood. Cyclooxygenase-2 overexpression causes hypoxia, oxidative stress, and endoplasmic reticulum stress. The aim of this study is to investigate the correlations among cyclooxygenase-2 expression, endoplasmic reticulum stress-associated molecules, and autophagy-associated molecules in triple-negative breast cancer. Surgical specimens from two cohorts of triple-negative breast cancer patients without neoadjuvant systemic therapy were analyzed: cohorts 1 and 2 consisted of 218 cases from 2004 to 2006 and 221 cases from 2007 to 2009, respectively. Specimens were evaluated by immunohistochemical examination of cyclooxygenase-2, endoplasmic reticulum stress markers, and autophagy markers expression using tissue microarrays. Cyclooxygenase-2 was overexpressed in 29.8% and 23.9% of cases in cohorts 1 and 2, respectively; and it was positively correlated with two out of three endoplasmic reticulum stress-associated molecules (XBP1, p = 0.025 and p = 0.003 in cohort 1 and cohort 2, respectively; PERK, p < 0.001 in both cohorts). Cyclooxygenase-2 was also positively correlated with two out of three autophagy markers (p62, p = 0.002 and p = 0.003 in cohort 1 and cohort 2, respectively; beclin1, p < 0.001 in both cohorts). Although cyclooxygenase-2 was not an independent prognostic factor for distant metastasis free survival and overall survival, its expression was associated with the expression of endoplasmic reticulum stress and autophagy molecules in triple-negative breast cancer.

## Introduction

Triple-negative breast cancer (TNBC) is a subtype of breast cancer that is characterized by the lack of expression of the estrogen and progesterone receptors as well as the lack of overexpression of human epidermal growth factor 2 (HER2). Although TNBC accounts for only approximately 10%–15% of all breast cancers, it is disproportionally lethal because of its aggressive behavior and absence of targeted therapies [1]. Many TNBC biomarkers (e.g., prognostic or predictive indicators) as well as novel therapies have been studied. These include targeted inhibitors and immune checkpoint inhibitors, which have been incorporated into the therapeutic regimen in some settings. For example, the importance of tumor-infiltrating lymphocytes as a candidate biomarker is increasing. However, cytotoxic chemotherapy still remains the mainstay therapy because of the heterogeneity within this single subtype of breast cancer. Therefore, the clinical demand for new TNBC treatment strategies remains urgent.

Given its high metabolic profile and aggressive nature, autophagy and the stress response are critical to the growth regulation of TNBC [2]. To maintain ER homeostasis during genotoxic stress, various cancers, including breast cancer, induce an unfolded protein response (UPR) and autophagy. Also, it is well known that all three UPR arms regulate autophagy [3]. Among the main responses of UPR, the IRE1α-XBP1 and PERK/eIF2α/ATF4 pathways play critical roles in TNBC, such as tumor initiation, vascularization, progression, and tumor microenvironment reprogramming [4, 5]. While there has been little evidence for a direct role for the ATF6 branch (another major pathway in the UPR response) in breast cancer, Sicari *et al*. found that ATF6 activity contributes to TNBC cells’ viability and invasion [6]. Autophagy-related proteins, including Beclin1 and LC3, are highly expressed in TNBC compared to other breast cancer subtypes due to the hypoxic conditions associated with the former. Knockdown of beclin1 and LC3 significantly decreases autophagy and inhibits proliferation, migration, and invasion of TNBC cell lines. Another major participant in autophagy is p62, whose accumulation is associated with advanced stage, positive lymph node and lymphovascular invasion in TNBC [7–9]. Collectively, mounting evidence suggests the importance of the UPR response and autophagy in promoting aggressiveness in TNBC. Nevertheless, no commercialized strategy for TNBC treatment has targeted these pathways.

Cyclooxygenase-2 (COX-2) is an inducible, proinflammatory enzyme that catalyzes the rate-limiting step in eicosanoid synthesis, i.e., the conversion of arachidonic acid to prostaglandins and thromboxanes. Numerous studies have highlighted the importance of COX-2 in tumor initiation, progression, invasion, multidrug resistance, and metastasis [10, 11]. Among the products of COX-2, prostaglandin E2 (PGE2) is thought to play a pivotal role in the pro-oncogenic action of COX-2. PGE2 inhibits apoptosis and promotes angiogenesis, proliferation, and metastasis [12, 13]. Although levels of COX-2 are tightly controlled by transcriptional and translational processes in most tissues, COX-2 is frequently overexpressed in breast cancers, particularly TNBC [14]. While early evidence suggests that the PGE2 pathways are important to the progression and chemotherapy resistance of TNBC, the exact mechanisms involved in the COX-2-mediated regulation of these processes are largely unknown.

In this study, we elucidated the correlations among COX-2 expression, endoplasmic reticulum (ER) stress-associated molecules, and autophagy-associated molecules in TNBC, including in patient-derived xenograft (PDX) models. We also measured tumor-infiltrating lymphocyte (TIL) levels in TNBC and its association with COX-2. Our objectives were to determine the associations between these known altered markers of TNBC, and to understand the mechanisms used by these proteins and pathways in the biological and clinical behavior of TNBC.

## Materials and methods

### Patients and tissue specimens

A total of 439 TNBC patients who underwent surgery for primary breast cancer between 2004 and 2009 at Asan Medical Center in Seoul, Korea were retrospectively selected. Of these 439 patients, cohorts 1 and 2 consisted of 218 patients who underwent surgery from 2004 to 2006 and 221 patients who underwent surgery from 2007 to 2009, respectively. Within cohorts 1 and 2, 141 (64.7%) and 153 (69.2%) patients, respectively, had no lymph node metastasis and received four cycles of adjuvant adriamycin (60 mg/m^2^) and cyclophosphamide (600 mg/m^2^). In the remaining cohorts 1 and 2 patients, 77 (35.3%) and 68 (30.8%) patients, respectively, presented with lymph node metastases and underwent four cycles of adriamycin and cyclophosphamide, followed by either four cycles of paclitaxel (175 mg/m^2^) or four cycles of docetaxel (75 mg/m^2^). Radiotherapy was performed on 348 patients (79.3%). The median follow-up time was 120.7 months. Clinicopathologic data, such as age at diagnosis, histologic grade, pathologic T stage, and N stage were obtained from medical records and pathology reports from the surgery. All procedures performed in studies involving human participants were performed in accordance with the Declaration of Helsinki. The need for consent has been waved by Institutional Review Board of Asan Medical Center (2013–0866).

### Histological evaluation

Representative tumor sections obtained through surgery were stained with hematoxylin and eosin and reviewed by two pathologists (H.J.L. and G.G.) to evaluate TIL levels (defined as the mean percentage of stroma in invasive carcinoma infiltrated by lymphocytes in 10% increments) [15], as well as histologic subtype and grade. Histologic types were identified based on the 2020 World Health Organization criteria, while the histologic grade was assessed using the modified Bloom-Richardson classification [16, 17].

### PDX model

Fresh tumor tissue samples obtained from TNBC patients from 2015 to 2020 were sliced into 2–3 mm^3^ fragments and grafted onto the inguinal mammary fat pad of four-week-old female NOD/SCID mice (KoaTech, Pyeongtaek, Gyeonggi-do, Korea). For tumor implantation, animals were anesthetized with 10mg/kg Alfaxan® (Jurox Pty Ltd, Australia) and 1.2mg/kg Rompun® (Bayer Korea, Korea) by intraperitoneal injection. After tumor engraftment, the mice were maintained on a 12-hour light/dark cycle in specific-pathogen-free (SPF) conditions and monitored twice a week for one year. Feed and water were freely provided for consumption. The tumor was excised when the xenograft tumor reached a size of 500 mm^2^.

The animal experiments were carried out ethically under protocols approved by the Institutional Animal Care and Use Committee of the Asan Institute for Life Sciences (2018-12-059). All experiments were performed under anaesthesia, and when the tumor was excised, the animals were euthanized in the chamber by adding a low concentration of CO_2_ and via subsequent cervical dislocation. All efforts were made to minimize suffering. The animals were managed under 24-hour surveillance by veterinarians and staff assigned to the facility under SPF conditions.

### Immunohistochemistry

Formalin-fixed, paraffin-embedded tissue samples were arrayed with a tissue-arraying instrument as previously described [18]. A tissue microarray was also constructed using 37 TNBC PDX tumors. Tissue microarray sections were stained using an automated immunohistochemical staining device (Benchmark XT; Ventana Medical Systems, Tucson, AZ). Antibodies targeting cyclooxygenase-2 (COX-2; 1:400, PA5-16817, ThermoFisher, Waltham, MA), phospho-eukaryotic initiation factor 2a (p-eIF2a; 1:200, ab32157, Abcam, Cambridge, UK), protein kinase RNA-like endoplasmic reticulum kinase (PERK; 1:200, Cell Signaling Technology, Danvers, MA), X-box binding protein-1 (XBP1; 1:75, ab37152, Abcam), Microtubule-associated protein 1A/1B-light chain 3 beta (LC3b; 1:200, ab48394, Abcam), p62 (1:10000, ab56416, Abcam), and beclin1 (1:50, ab51031, Abcam) were used. The nuclear and cytoplasmic expression levels of XBP1, LC3B, p62, beclin1 were evaluated separately. The intensity of immunochemical staining was graded as 0 (negative), 1 (weak), 2 (moderate), or 3 (strong), and the percentage of positive tumor cells was also evaluated. The “immunoreactive score” was calculated by multiplying the percentage of coverage by the staining intensity.

### Statistical analysis

All statistical analyses were performed using SPSS statistical software ver. 26.0 (IBM Corp., Armonk, NY, USA). Mann–Whitney U test, Spearman’s correlation analysis, the chi-square test, Cox proportional hazards regression model were used as appropriate. All statistical analyses were two-sided, and statistical significance was set at p < 0.05.

## Results

### Clinicopathologic characteristics

All 439 patients were female, with a median age of 47 years at diagnosis (range, 23 to 76 years). Histologic grades of 2 and 3 were scored in 102 (23.2%) and 337 (76.8%) cases, respectively. Tumors consisted of 180 pT1 (41.0%), 238 pT2 (54.2%), and 21 pT3 (4.8%) tumors. No pT4 tumors were detected. Most (n = 294) of the patients did not have pathologic lymph node metastasis (pN0, 67.0%), while 74 (17.0%), 36 (8.2%), and 35 (8.0%) tumors were pN1, pN2, and pN3, respectively. The tumors were categorized into four groups based on TIL amounts: <10% TILs (100 patients, 22.8%), ≥10%; <30% TILs (99 patients, 22.6%), ≥30%; and <60% TILs (99 patients, 22.6%), ≥60% TILs (141 patients, 32.1%). The clinicopathologic parameters of cohorts 1 and 2 did not differ significantly (Table 1).

**Table 1:** Comparison of clinicopathologic variables of cohorts 1 and 2.

	Cohort 1	Cohort 2	p-value
**Age**			
<50	134 (61.5)	135 (61.1)	0.935
≥50	84 (38.5)	86 (38.9)	
**Histologic grade**			
1 and 2	57 (26.1)	45 (20.4)	0.151
3	161 (73.9)	176 (79.6)	
**pT**			
1	87 (39.9)	93 (42.1)	0.863
2	121 (55.5)	117 (52.9)	
3	10 (4.6)	11 (5.0)	
**TIL**			
<10%	52 (23.9)	48 (21.7)	0.524
≥10% and <30%	53 (24.3)	46 (20.8)	
30% and <60%	50 (22.9)	49 (22.2)	
≥60%	63 (28.9)	78 (35.3)	
**LN metastasis**			
Negative	141 (64.7)	153 (69.2)	0.311
Positive	77 (35.3)	68 (30.8)	

**pT**, pathological tumor stage; **TIL**, tumor-infiltrating lymphocyte; **LN**, lymph node

### Expression of COX-2

**Fig 1:**
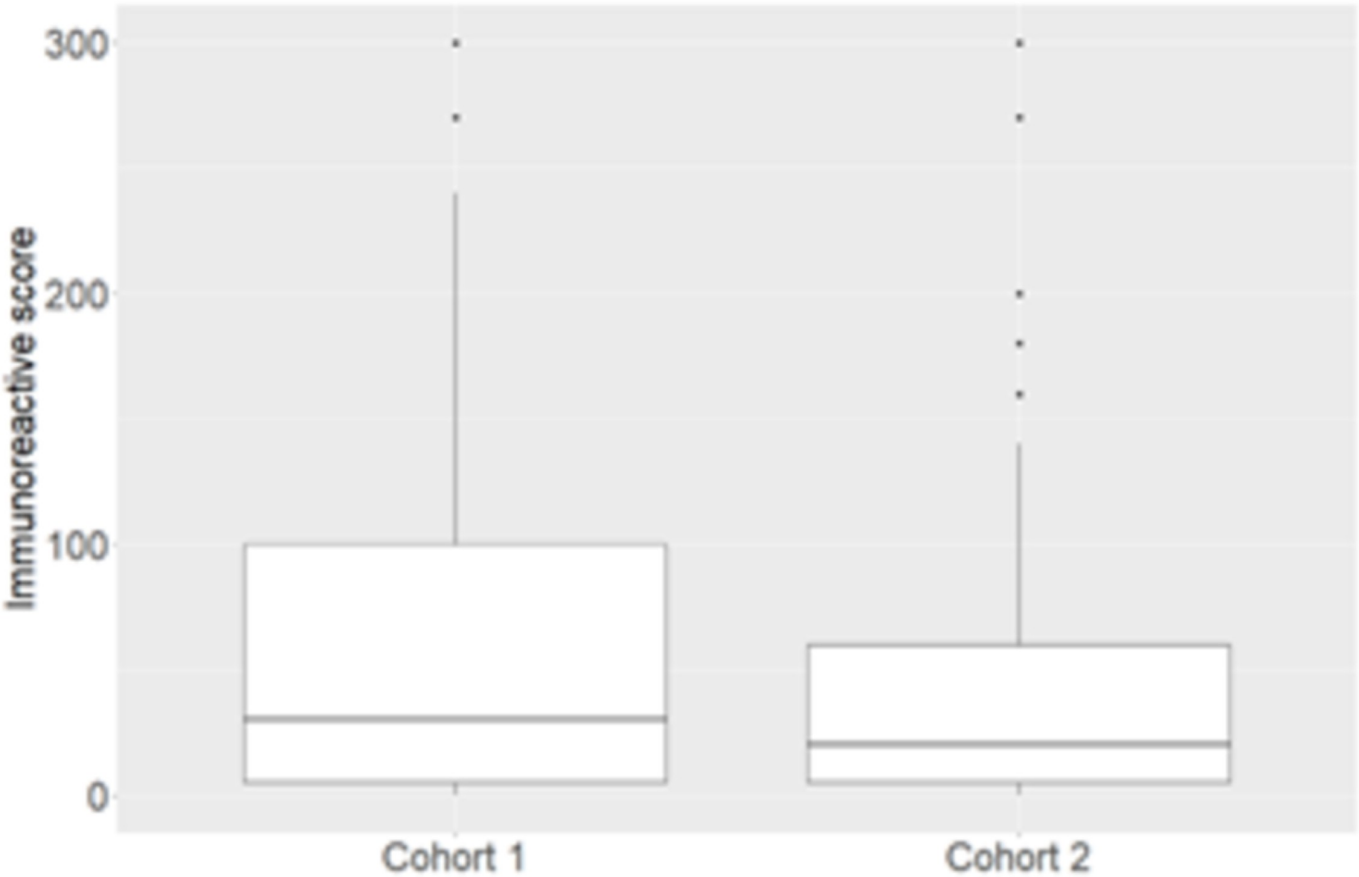
Distribution of triple-negative breast cancer patients in cohorts 1 and 2 based on immunoreactive scores for COX-2 expression.

The COX-2 immunoreactive scores of the two cohorts did not differ significantly (p = 0.072, Fig 1). The average value of the sum of COX-2 immunoreactive scores of both cohorts was 64.9. Therefore, immunohistochemical analysis of COX-2 expression was scored as follows: below average, low expression (321 cases, 73.1%); and above average, high expression (118 cases, 26.8%). In cohort 1, 153 cases (70.1%) were low and 65 cases (29.8%) were high; while in cohort 2, 168 cases (76.0%) were low and 53 cases (23.9%) were high. Meanwhile, the average COX-2 immunoreactive score among successful engraftments for the PDX model was 220.5, which is significantly higher than that of primary tumors.

### Characteristics of tumors according to COX-2 expression

In cohort 1, high COX-2 expression was significantly associated with lower TIL levels (p = 0.002). In cohort 2, a trend of association was seen between the TIL level and COX-2 expression (p = 0.087). COX-2 expression levels were not significantly associated with age, histologic grade, pT stage, and lymph node metastasis in both TNBC cohorts (Table 2).

**Table 2:** Comparison of clinicopathologic variables according to COX-2 expression level.

	COX-2 immunoreactive score (Cohort 1)			COX-2 immunoreactive score (Cohort 2)		
	Low (%)	High (%)	p-value	Low (%)	High (%)	p-value
**Age**						
<50	98 (64.1)	36 (55.4)	0.229	106 (63.1)	29 (54.7)	0.275
≥50	55 (35.9)	29 (44.6)		62 (36.9)	24 (45.3)	
**Histologic grade**						
1 and 2	36 (23.5)	21 (32.3)	0.177	31 (18.5)	14 (26.4)	0.209
3	117 (76.5)	44 (67.7)		137 (81.5)	39 (73.6)	
**pT**						
1	64 (41.8)	23 (35.4)	0.572	70 (41.7)	23 (43.4)	0.562
2	83 (54.2)	38 (58.5)		91 (54.2)	26 (49.1)	
3	6 (3.9)	4 (6.2)		7 (4.2)	4 (7.5)	
**LN metastasis**						
Negative	100 (65.4)	41 (63.1)	0.747	116 (69.0)	37 (69.8)	0.916
Positive	53 (34.6)	24 (36.9)		52 (31.0)	16 (30.2)	
**TIL**						
<10%	27 (17.6)	25 (38.5)	0.001	32 (19.0)	16 (30.2)	0.087
≥10% and <30%	35 (22.9)	18 (27.7)		33 (19.6)	13 (24.5)	
30% and <60%	40 (26.1)	10 (15.4)		41 (24.4)	8 (15.1)	
≥60%	51 (33.3)	12 (18.5)		62 (36.9)	16 (30.2)	

**pT**, pathological tumor stage; **LN**, lymph node; **TIL**, tumor-infiltrating lymphocyte

### Correlation of COX-2 expression with ER stress and autophagy markers

**Fig 2:**
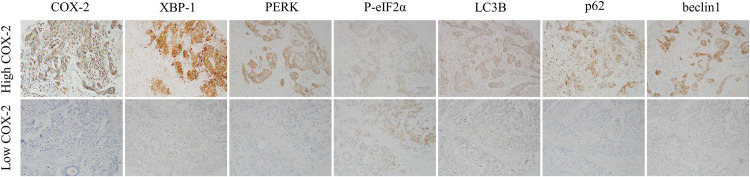
Representative immunohistochemical staining images of ER stress and autophagy markers according to COX-2 expression (×200) Tumors with high COX-2 expression levels also show high levels of ER stress (XBP-1 and PERK) and autophagy (p62, beclin1) markers. In contrast, tumors with low COX-2 expression levels show low levels of ER stress (XBP-1 and PERK) and autophagy (p62, beclin1) markers.

ER stress is known to induce the expression of COX-2 [19]; moreover, NSAIDs, including celecoxib, modulate autophagy [20, 21]. Therefore, we first analyzed the cytoplasmic association among the COX-2 immunoreactive scores, ER stress-associated molecules, and autophagy markers. We observed positive correlations between COX-2 and two out of three ER stress-associated molecules: XBP1 (p = 0.025 in cohort 1 and p = 0.003 in cohort 2), PERK (p < 0.001 in both cohorts), and p-eIF2a (p > 0.05 in both cohorts). COX-2 was also positively correlated with two out of three autophagy markers in both cohorts: p62 (p = 0.002 in cohort 1 and p = 0.003 in cohort 2), beclin1 (p < 0.001 in both cohorts), and LC3B (p > 0.05 in both cohorts) (Figs 2 and S1 and S2).

In the 37 TNBC PDX models, COX-2 was also positively correlated with the same ER stress-associated molecules: XBP1 (r = 0.560, p < 0.001), PERK (r = 0.591, p < 0.001), and p-eIF2a (r = 0.286, p = 0.086); however, it was positively correlated with only one autophagy-related molecule, p62 (r = 0.606, p < .001) (S1 Table).

### Prognostic significance of COX-2 expression

While the overexpression of COX-2 is known to be a poor prognostic marker in breast cancer, COX-2 expression is higher in TNBC than in other subtypes. Therefore, we analyzed the prognostic significance of COX-2 expression in two TNBC cohorts. Univariate and multivariate analyses of age, pT stage, histologic grade, TIL level, and lymph node involvement were undertaken to elucidate the prognostic significance of COX-2 expression. Univariate analysis showed that TIL level and lymph node involvement were significantly associated with overall survival (OS) in both cohorts; while pT stage, TIL, and lymph node involvement were significantly associated with distant metastasis free survival (DMFS) in both cohorts (Table 3). Multivariate analysis found that only TIL level was an independent prognostic factor for OS in both cohorts, while TIL and lymph node involvement were independent prognostic factors for DMFS in both cohorts. Contrary to our expectation, COX-2 expression was not significantly associated with OS in both cohorts (in univariate analysis, p = 0.441 in cohort 1 and p = 0.190 in cohort 2; in multivariate analysis, p = 0.924 in cohort 1 and p = 0.115 in cohort 2). Interestingly, COX-2 expression was significantly associated with DMFS in only cohort 1, based on univariate analysis (p = 0.002 in cohort 1 and p = 0.145 in cohort 2). However, multivariate analysis revealed that COX-2 expression was not an independent prognostic factor for DMFS (p = 0.056 in cohort 1 and p = 0.089 in cohort 2). Therefore, COX-2 expression was not an independent prognostic factor for OS and DMFS.

**Table 3:** Univariate and multivariate analyses of overall survival and distant metastasis free survival in each cohort.

	Cohort 1 (N = 218)				Cohort 2 (N = 221)			
	Univariate HR (95% CI)	p value	Multivariate HR (95% CI)	Multivariate p	Univariate HR (95% CI)	p value	Multivariate HR (95% CI)	Multivariate p
**Overall survival**	**Age (<50 vs. ≥50 years)**							
	1.345	0.327	1.147	0.657	1.106	0.764	1.135	0.714
	(0.743–2.435)		(0.625–2.105)		(0.574–2.132)		(0.576–2.239)	
	**pT stage (≤5 cm vs. >5 cm)**							
	2.637	0.065	2.254	0.13	3.199	0.028	2.013	0.222
	(0.943–7.376)		(0.786–6.460)		(1.133–9.034)		(0.656–6.180)	
	**Histological grade (Grade 3 vs. grade 1 and 2)**							
	1.063	0.861	1.35	0.41	1.381	0.469	1.515	0.361
	(0.537–2.103)		(0.661–2.757)		(0.576–3.311)		(0.621–3.693)	
	**TIL (percentage)**							
	0.984	0.015	0.983	0.013	0.982	0.006	0.979	0.002
	(0.972–0.997)		(0.969–0.996)		(0.970–0.995)		(0.966–0.992)	
	**Node (negative vs. positive)**							
	1.829	0.046	1.785	0.061	2.677	0.003	2.515	0.009
	(1.012–3.306)		(0.973–3.273)		(1.405–5.103)		(1.261–5.016)	
	**COX–2 expression**							
	1.001	0.441	1	0.924	0.997	0.19	0.996	0.115
	(0.998–1.005)		(0.997–1.003)		(0.992–1.002)		(0.992–1.001)	
**Distant metastasis free survival**	**Age (<50 vs. ≥50 years)**							
	0.984	0.965	0.72	0.388	1.168	0.67	1.219	0.598
	(0.478–2.027)		(0.341–1.519)		(0.572–2.384)		(0.584–2.547)	
	**pT stage (≤5 cm vs. >5 cm)**							
	3.908	0.011	3.242	0.035	3.838	0.012	2.188	0.182
	(1.367–11.175)		(1.085–9.685)		(1.342–1.978)		(0.694–6.902)	
	**Histological grade (Grade 3 vs. grade 1 and 2)**							
	0.732	0.418	1.231	0.607	1.092	0.846	1.157	0.755
	(0.345–1.555)		(0.557–2.723)		(0.448–2.663)		(0.464–2.882)	
	**TIL (percentage)**							
	0.969	0.001	0.97	0.003	0.983	0.014	0.98	0.007
	(0.951–0.987)		(0.951–0.990)		(0.969–0.996)		(0.966–0.994)	
	**Node (negative vs. positive)**							
	2.72	0.006	2.264	0.032	3.082	0.002	2.897	0.006
	(1.332–5.553)		(1.074–4.771)		(1.518–6.255)		(1.348–6.227)	
	**COX–2 expression**							
	1.005	0.002	1.003	0.056	0.996	0.145	0.995	0.089
	(1.002–1.009)		(1.000–1.007)		(0.990–1.001)		(0.990–1.001)	

**HR**, hazard ratio; **pT**, pathological tumor stage; **TIL**, tumor-infiltrating lymphocyte

## Discussion

To the best of our knowledge, this study is the first to explore the correlation between COX-2, ER stress, and autophagy in TNBC using protein assays. We found that higher COX-2 levels in TNBC were associated with higher ER stress and autophagy markers. Analysis of TNBC PDX models showed not only the same correlation patterns as those found in the primary tumor, but these models also detected a much larger correlation coefficient (Figs 2 and S1 and S2). These results suggest the possibility that COX-2 may be a part of the cell stress response, because TNBCs, which are notably aggressive tumors, have a high PDX success rate [22]. COX-2 mediates the interaction between heavy metal-induced ER stress and autophagy via the eIF2α–ATF4 pathway, a major autophagy pathway [23, 24]. In the non-TNBC pre-clinical models MCF-7 and ML-1, both COX-2 and autophagy pathways are activated upon ER stress [19], which has also been reported in TNBC MDA-MB-231 cell lines [25]. In non-breast cancer model chondrocytes, increased ER stress is associated with increased COX-2 expression [26]. Moreover, the relationship between ER stress and autophagy has been well established. Interestingly, three branches of the UPR pathway are linked to autophagy-related signaling pathways [27] as part of critical cell stress response regulation mechanisms. Yet, the exact contribution of COX-2 to these cell stress pathways in TNBC remain unclear.

Considering the results of previous reports on the associations among COX-2, ER stress, and autophagy pathways, we hypothesize that the correlation observed in the current study are a result of the high metabolic activity and aggressive nature of TNBC. Cells affected by TNBC are well known to undergo intense and wasteful metabolic activities, resulting in activation of both autophagy and ER stress pathways. However, to date, the involvement between COX-2 and cell stress response is unknown. Our current study is purely observational, thus further mechanistic studies are needed to determine the mechanisms of action of COX-2, ER stress, and autophagy under high stress conditions similar to those of TNBC. However, we are confident that our study has shed new insight on the role of COX-2 in cell stress responses through direct analysis of patient samples.

It is known that COX-2 levels in breast cancers are elevated, ranging from 27.9% to 81.4%. These overexpression levels are highly variable because of the differences in scoring methods, staining protocols, and cut-off points among different studies. Interestingly, basal-like TNBC is associated with COX-2 overexpression [14, 28, 29]. COX-2 overexpression is known to be a poor prognostic factor in breast cancer. Although reports of the prognostic value of COX-2 vary from study to study, and lead to no consensus, a recent meta-analysis by Xu *et al*. [28] showed that COX-2 overexpression in breast cancer is associated with poor prognosis as well as larger tumor size and lymph node metastasis. However, few studies have addressed the association between COX-2 level and prognosis of TNBC patients, and regarding breast cancer, there is no consensus yet. Zhou *et al*. [30] reported that COX-2 overexpression is significantly correlated with a shorter OS time in 31 TNBC patients. Similarly, Chikman *et al*. [31] reported that COX-2 is associated with a decreased DFS in a cohort of 67 TNBC patients. In contrast, Kwon *et al*. [32] failed to show the clinical significance of COX-2 in TNBC patients. In our study, univariate analysis of cohort 1 showed that COX-2 was a poor prognostic factor for DMFS, but COX-2 did not act as an independent prognostic factor of TNBC because it was observed only in univariate analysis. Moreover, COX-2 also showed no significant relationship to OS. Our study, consistent with the results of other studies, failed to demonstrate that COX-2 can act as an independent predictor of any type of patient survival in TNBC. Nevertheless, additional studies are required, considering that other studies have been based on small cohorts.

Association of COX-2 expression in invasive tumor cells and the level of TILs has not been analyzed previously. In this study, we found a negative correlation of these two factors in both cohorts. Cohort 1 showed a statistically significant correlation, but cohort 2 showed only a trend of association. These associations need to be further investigated.

Our study found that cancers with higher COX-2 expression levels also showed elevated ER stress and autophagy marker levels. Based on these results, we hypothesize that the COX-2 is the actual promoter of these pathways, and therefore a suppression of COX-2 by therapeutic intervention (e.g., COX-2 inhibitor celecoxib) may mitigate activated cell stress response. In theory, attenuation of the cell stress response will result in the stress-induced death of cancer cells. Although further studies on this treatment regimen are needed, a useful approach may be to examine COX-2 levels in TNBC patients treated with celecoxib. Indeed, anti-COX-2 agents such as celecoxib have been tested in clinical trials [33]. More in depth mechanistic studies will provide further insight into the appropriate direction of therapeutic strategies for one of the most aggressive breast cancers, TNBC.

## Supporting information

S1 Fig Correlations between the immunoreactive scores of COX-2, ER stress, and autophagy markers in cohort 1. (*p < 0.05, **p < 0.01, ***p < 0.001). (PDF)

S2 Fig Correlations between the immunoreactive scores of COX-2, ER stress, and autophagy markers in cohort 2. (*p < 0.05, **p < 0.01, ***p < 0.001).
(PDF)

S1 Table Correlations between the immunoreactive scores of COX-2, ER stress, and autophagy markers in PDX models. (PDF)

## References

[1] Dent R, Trudeau M, Pritchard KI, Pritchard KI, Hanna WM, Kahn HK, et al. Triple-negative breast cancer: clinical features and patterns of recurrence. Clin Cancer Res. 2007;13(15 Pt 1):4429–4434. 10.1158/1078-0432.CCR-06-3045 17671126

[2] Abd El-Aziz YS, Gillson J, Jansson PJ, Sahni S. Autophagy: A promising target for triple negative breast cancers. Pharmacol Res. 2022;175:106006. 10.1016/j.phrs.2021.106006 34843961

[3] Hoyer-Hansen M, Jaattela M. Connecting endoplasmic reticulum stress to autophagy by unfolded protein response and calcium. Cell Death Differ. 2007;14(9):1576–1582. 10.1038/sj.cdd.4402200 17612585

[4] McGrath EP, Logue SE, Mnich K, Deegan S, Jäger R, Gorman AM, et al. The unfolded protein response in breast cancer. Cancers (Basel). 2018;10(10). 10.3390/cancers10100344 30248920 PMC6211039

[5] Harnoss JM, Le Thomas A, Reichelt M, Guttman O, Wu TD, Marsters SA, et al. IRE1alpha Disruption in triple-negative breast cancer cooperates with antiangiogenic therapy by reversing ER stress adaptation and remodeling the tumor microenvironment. Cancer Res. 2020;80(11):2368–2379. 10.1158/0008-5472.CAN-19-3108 32265225 PMC7272310

[6] Sicari D, Fantuz M, Bellazzo A, Valentino E, Apollonio M, Pontisso I, et al. Mutant p53 improves cancer cells’ resistance to endoplasmic reticulum stress by sustaining activation of the UPR regulator ATF6. Oncogene. 2019;38(34):6184–6195. 10.1038/s41388-019-0878-3 31312025

[7] Choi J, Jung W, Koo JS. Expression of autophagy-related markers beclin-1, light chain 3A, light chain 3B and p62 according to the molecular subtype of breast cancer. Histopathology. 2013;62(2):275–286. 10.1111/his.12002 23134379

[8] Hamurcu Z, Delibasi N, Gecene S, Şener EF, Dönmez-Altuntaş H, Özkul Y, et al. Targeting LC3 and Beclin-1 autophagy genes suppresses proliferation, survival, migration and invasion by inhibition of Cyclin-D1 and uPAR/Integrin beta1/ Src signaling in triple negative breast cancer cells. J Cancer Res Clin Oncol. 2018;144(3):415–430. 10.1007/s00432-017-2557-5 29288363 PMC11813384

[9] Luo RZ, Yuan ZY, Li M, Xi SY, Fu J, He J. Accumulation of p62 is associated with poor prognosis in patients with triple-negative breast cancer. Onco Targets Ther. 2013;6:883–888. 10.2147/OTT.S46222 23888115 PMC3722135

[10] Li S, Jiang M, Wang L, Yu S. Combined chemotherapy with cyclooxygenase-2 (COX-2) inhibitors in treating human cancers: Recent advancement. Biomed Pharmacother. 2020;129:110389. 10.1016/j.biopha.2020.110389 32540642

[11] Tong D, Liu Q, Wang LA, Xie Q, Pang J, Huang Y, et al. The roles of the COX2/PGE2/EP axis in therapeutic resistance. Cancer Metastasis Rev. 2018;37(2–3):355–368. 10.1007/s10555-018-9752-y 30094570

[12] Wu WK, Sung JJ, Lee CW, Yu J, Cho CH. Cyclooxygenase-2 in tumorigenesis of gastrointestinal cancers: an update on the molecular mechanisms. Cancer Lett. 2010;295(1):7–16. 10.1016/j.canlet.2010.03.015 20381235

[13] Goswami S, Sharma-Walia N. Crosstalk between osteoprotegerin (OPG), fatty acid synthase (FASN) and, cycloxygenase-2 (COX-2) in breast cancer: implications in carcinogenesis. Oncotarget. 2016;7(37):58953–58974. 10.18632/oncotarget.9835 27270654 PMC5312288

[14] Mosalpuria K, Hall C, Krishnamurthy S, Lodhi A, Hallman DM, Baraniuk MS, et al. Cyclooxygenase-2 expression in non-metastatic triple-negative breast cancer patients. Mol Clin Oncol. 2014;2(5):845–850. 10.3892/mco.2014.327 25054056 PMC4106732

[15] Salgado R, Denkert C, Demaria S, Sirtaine N, Klauschen F, Pruneri G, et al. The evaluation of tumor-infiltrating lymphocytes (TILs) in breast cancer: recommendations by an International TILs Working Group 2014. Ann Oncol. 2015;26(2):259–271. 10.1093/annonc/mdu450 25214542 PMC6267863

[16] Lakhani SR, Ellis IO, Schnitt S, Tan PH, van de Vijver M. WHO Classification of Tumours of the Breast. 2012.

[17] Elston CW, Ellis IO. Pathological prognostic factors in breast cancer. I. The value of histological grade in breast cancer: experience from a large study with long-term follow-up. Histopathology. 1991;19(5):403–410. 10.1111/j.1365-2559.1991.tb00229.x 1757079

[18] Lee HJ, Seo AN, Park SY, Kim JY, Park JY, Yu JH, et al. Low prognostic implication of fibroblast growth factor family activation in triple-negative breast cancer subsets. Ann Surg Oncol. 2014;21(5):1561–1568. 10.1245/s10434-013-3456-x 24385208

[19] Hung JH, Su IJ, Lei HY, Wang HC, Lin WC, Chang WT, et al. Endoplasmic reticulum stress stimulates the expression of cyclooxygenase-2 through activation of NF-kappaB and pp38 mitogen-activated protein kinase. J Biol Chem. 2004;279(45):46384–46392. 10.1074/jbc.M403568200 15319438

[20] Fu X, Tan T, Liu P. Regulation of autophagy by non-steroidal anti-inflammatory drugs in cancer. Cancer Manag Res. 2020;12:4595–4604. 10.2147/CMAR.S253345 32606952 PMC7305821

[21] Thomas S, Sharma N, Golden EB, Cho H, Agarwal P, Gaffney KJ, et al. Preferential killing of triple-negative breast cancer cells in vitro and in vivo when pharmacological aggravators of endoplasmic reticulum stress are combined with autophagy inhibitors. Cancer Lett. 2012;325(1):63–71. 10.1016/j.canlet.2012.05.030 22664238

[22] Jung J, Seol HS, Chang S. the generation and application of patient-derived xenograft model for cancer research. Cancer Res Treat. 2018;50(1):1–10. 10.4143/crt.2017.307 28903551 PMC5784646

[23] Luo B, Lin Y, Jiang S, Huang L, Yao H, Zhuang Q, et al. Endoplasmic reticulum stress eIF2alpha-ATF4 pathway-mediated cyclooxygenase-2 induction regulates cadmium-induced autophagy in kidney. Cell Death Dis. 2016;7(6):e2251. 10.1038/cddis.2016.78 27253415 PMC5143407

[24] Chen P, Geng N, Zhou D, Zhu Y, Xu Y, Liu K, et al. The regulatory role of COX-2 in the interaction between Cr(VI)-induced endoplasmic reticulum stress and autophagy in DF-1 cells. Ecotoxicol Environ Saf. 2019;170:112–119. 10.1016/j.ecoenv.2018.11.120 30529609

[25] Cheng X, Liu H, Jiang CC, Fang L, Chen C, Zhang XD, et al. Connecting endoplasmic reticulum stress to autophagy through IRE1/JNK/beclin-1 in breast cancer cells. Int J Mol Med. 2014;34(3):772–781. 10.3892/ijmm.2014.1822 24970676

[26] Rasheed Z, Haqqi TM. Endoplasmic reticulum stress induces the expression of COX-2 through activation of eIF2alpha, p38-MAPK and NF-kappaB in advanced glycation end products stimulated human chondrocytes. Biochim Biophys Acta. 2012;1823(12):2179–2189. 10.1016/j.bbamcr.2012.08.021 22982228 PMC4509732

[27] Lin Y, Jiang M, Chen W, Zhao T, Wei Y. Cancer and ER stress: Mutual crosstalk between autophagy, oxidative stress and inflammatory response. Biomed Pharmacother. 2019;118:109249. 10.1016/j.biopha.2019.109249 31351428

[28] Xu F, Li M, Zhang C, Cui J, Liu J, Li J, et al. Clinicopathological and prognostic significance of COX-2 immunohistochemical expression in breast cancer: a meta-analysis. Oncotarget. 2017;8(4):6003–6012. 10.18632/oncotarget.13990 27999206 PMC5351608

[29] Tian J, Hachim MY, Hachim IY, Dai M, Lo C, Raffa FA, et al. Cyclooxygenase-2 regulates TGFbeta-induced cancer stemness in triple-negative breast cancer. Sci Rep. 2017;7:40258. 10.1038/srep40258 28054666 PMC5215509

[30] Zhou L, Li K, Luo Y, Tian L, Wang M, Li C, et al. Novel prognostic markers for patients with triple-negative breast cancer. Hum Pathol. 2013;44(10):2180–2187. 10.1016/j.humpath.2013.03.021 23845466

[31] Chikman B, Vasyanovich S, Lavy R, Habler L, Tolstov G, Kapiev A, et al. COX2 expression in high-grade breast cancer: evidence for prognostic significance in the subset of triple-negative breast cancer patients. Med Oncol. 2014;31(6):989. 10.1007/s12032-014-0989-1 24816739

[32] Kwon J, Eom KY, Koo TR, Kim BH, Kang E, Kim SW, et al. A prognostic model for patients with triple-negative breast cancer: importance of the modified nottingham prognostic index and age. J Breast Cancer. 2017;20(1):65–73. 10.4048/jbc.2017.20.1.65 28382096 PMC5378581

[33] Coombes RC, Tovey H, Kilburn L, Mansi J, Palmieri C, Bartlett J, et al. Effect of celecoxib vs placebo as adjuvant therapy on disease-free survival among patients with breast cancer: The REACT Randomized Clinical Trial. JAMA Oncol. 2021;7(9):1291–1301. 10.1001/jamaoncol.2021.2193 34264305 PMC8283666

