# Supplementary material for: Association of cyclooxygenase-2 expression with endoplasmic reticulum stress and autophagy in triple-negative breast cancer
Source: PLoS One. 2023 Aug 4;18(8):e0289627. doi: 10.1371/journal.pone.0289627 (PMC10403079; doi:10.1371/journal.pone.0289627)
Supplement: S1 Fig — (PDF) [file pone.0289627.s001.pdf]

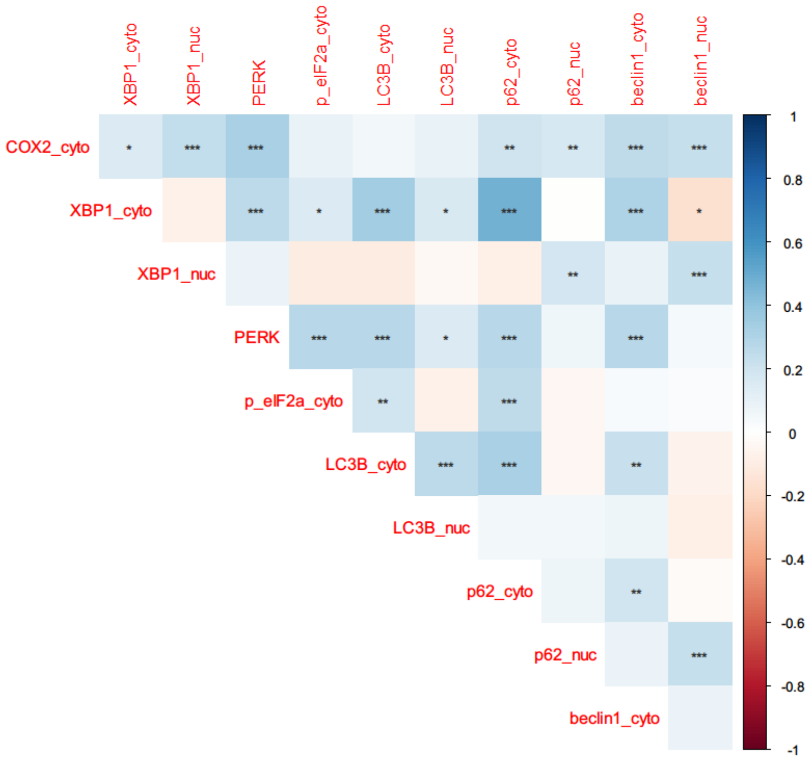

S1 Fig. Correlations between the immunoreactive scores of COX-2 and ER stress and autophagy markers in cohort 1(*$p < 0.05$, **$p < 0.01$, ***$p < 0.001$)**.**
