# Supplementary material for: Association of cyclooxygenase-2 expression with endoplasmic reticulum stress and autophagy in triple-negative breast cancer
Source: PLoS One. 2023 Aug 4;18(8):e0289627. doi: 10.1371/journal.pone.0289627 (PMC10403079; doi:10.1371/journal.pone.0289627)
Supplement: S1 Table — (PDF) [file pone.0289627.s003.pdf]

S1 Table. Correlations between the immunoreactive scores of COX-2 and ER stress and autophagy markers in PDX models

PDX		XBP1	PERK	p_eIF2a	LC3B	p62	Beclin1
COX-2	rho	**0.560**	**0.591**	0.286	0.287	**0.606**	0.184
	p	**<0.001**	**<0.001**	0.086	0.085	**<0.001**	0.275
XBP1	rho		**0.394**	**0.542**	0.271	**0.588**	0.197
	p		**0.016**	**0.001**	0.104	**<0.001**	0.243
PERK	rho			**0.484**	**0.602**	**0.612**	0.193
	p			**0.002**	**<0.001**	**<0.001**	0.251
p_eIF2a	rho				**0.442**	**0.661**	0.265
	p				**0.006**	**<0.001**	0.113
LC3b	rho					**0.408**	-0.089
	p					**0.012**	0.599
p62	rho						0.149
	p						0.379
